# PRESTOapp for health workers with mental health symptoms related to the COVID-19 pandemic

**DOI:** 10.1192/j.eurpsy.2022.1474

**Published:** 2022-09-01

**Authors:** M. Primé Tous, G. Anmella, X. Segú, M.D.R. Fernández Canseco, C. Carrino, M. Villegas, V. Vicens, J. Blanch, M. Cavero, E. Vieta, D. Hidalgo-Mazzei

**Affiliations:** 1 Hospital Clínic de Barcelona, Department Of Psychiatry And Psychology, Barcelona, Spain; 2 Barcelona Supercomputing Center, Text Mining Unit, Barcelona, Spain; 3 Abi Global Health, Chief Medical Officer And Co-founder, Barcelona, Spain; 4 Hospital Clínic,, 1department Of Psychiatry And Psychology, Barcelona, Spain; 5 Hospital Clínic de Barcelona, Bipolar And Depressive Disorders Unit, Institute Of Neuroscience, Barcelona, Spain

**Keywords:** Covid-19, Natural Language Processing (NLP), health workers, e-mental health

## Abstract

**Introduction:**

The COVID-19 pandemic has caused a significant impact on the mental health of health workers that has brought many hospitals to launch immediate preventive mental health programs.

**Objectives:**

(1) To adapt and enhance a smartphone app (PRESTOapp) for health workers with mental health symptoms related to the COVID-19, and (2) to demonstrate its potential effectiveness in significantly reducing anxiety-depressive and PTSD symptoms in this population. We aim to incorporate Natural Language Processing (NLP)-based techniques in a chatbot user-interface that will enable a more personalized and accurate monitoring and intervention.

**Methods:**

An 18-months study with a 6-months preliminary phase to adapt PRESTOapp to health workers, enhance it with NLP-based techniques and chatbot user-interface, and evaluate its feasibility, and effectiveness in 12-months.

**Results:**

PRESTOapp has the potential to provide a prompt, personalized and integral response to the mental health demand due to the COVID-19. It will help by providing an innovative digital platform, that will allow remote monitoring of the symptoms course, provide brief psychotherapeutic interventions, and detect urgent situations. If the preliminary results of this study point to a potential effectiveness of the intervention, PRESTOapp may be easily adapted to the general population.

**Conclusions:**

PRESTOapp may be one of the key digital platforms that may help preventing and treating potentially severe mental health consequences. Considering the unresolved problem of burnout in health workers even before the COVID-19, this project will develop the necessary technology for implementing cost-effective mental health solutions, not only during the pandemic.

**Disclosure:**

No significant relationships.

